# Comprehensive multiomics analyses reveal pervasive involvement of aberrant cohesin binding in transcriptional and chromosomal disorder of cancer cells

**DOI:** 10.1016/j.isci.2023.106908

**Published:** 2023-05-19

**Authors:** Jiankang Wang, Ryuichiro Nakato

**Affiliations:** 1 School of Biomedical Sciences, Hunan University, Changsha, China; 2 Institute for Quantitative Biosciences, The University of Tokyo, Bunkyo-ku, Tokyo, Japan

**Keywords:** Epigenetics, Cancer systems biology, Omics

## Abstract

Chromatin organization, whose malfunction causes various diseases including cancer, is fundamentally controlled by cohesin. While cancer cells have been found with mutated or misexpressed cohesin genes, there is no comprehensive survey about the presence and role of abnormal cohesin binding in cancer cells. Here, we systematically identified ∼1% of cohesin-binding sites (701–2,633) as cancer-aberrant binding sites of cohesin (CASs). We integrated CASs with large-scale transcriptomics, epigenomics, 3D genomics, and clinical information. CASs represent tissue-specific epigenomic signatures enriched for cancer-dysregulated genes with functional and clinical significance. CASs exhibited alterations in chromatin compartments, loops within topologically associated domains, and *cis*-regulatory elements, indicating that CASs induce dysregulated genes through misguided chromatin structure. Cohesin depletion data suggested that cohesin binding at CASs actively regulates cancer-dysregulated genes. Overall, our comprehensive investigation suggests that aberrant cohesin binding is an essential epigenomic signature responsible for dysregulated chromatin structure and transcription in cancer cells.

## Highlights

• About 1% cohesin-binding sites are identified as cancer aberrant sites (CASs)
• CASs are enriched for dysregulated genes with functional and clinical significance
• CASs are associated with dysregulated chromatin structure and gene regulation

## Graphical abstract

**Figure:**
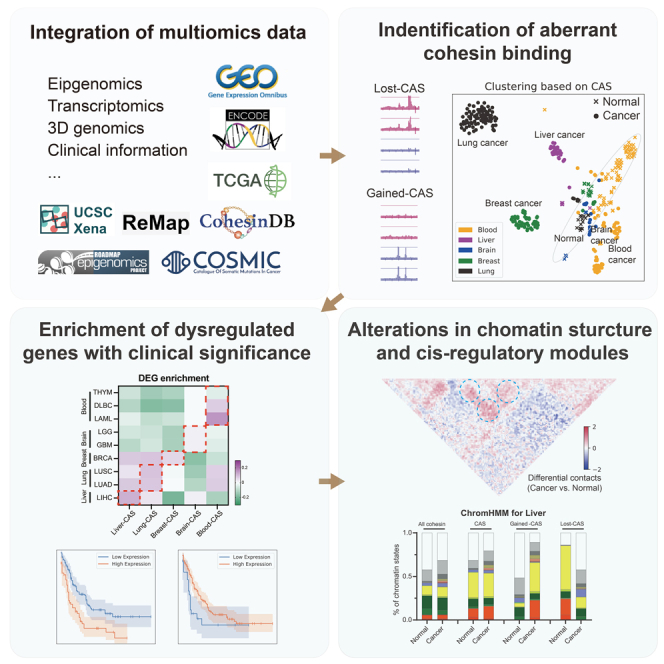

## Introduction

The human genome is precisely organized within the nucleus in a hierarchical three-dimensional (3D) manner.^1^ Within chromosome territories, chromosomes are organized into compartments A and B, which are associated with active and inactive self-interacting regions, respectively.^2^ At a higher structural level, topologically associated domains (TADs) describe submegabase domains, while chromatin loops provide finer resolution contact information.^3^^,^^4^ Incorrect chromosome structure misdirects the physical contacts between genomic loci, constituting pathogenic mechanisms that control various diseases, including cancer.^5^ For instance, perturbations of insulated TAD boundaries are thought to be sufficient to activate oncogenes;^6^^,^^7^ reprogramming of the multiscale 3D genome was found to control the transcriptome in prostate cancer^8^ and pancreatic cancer.^9^ Essentially, chromosome structure is regulated by several protein factors, abnormalities in which can cause genome misfolding and hence cancer.^10^ Cohesin, a ring-shaped chromosome-bound protein complex, is one such factor that fundamentally controls chromatin structure and transcription.^11^^,^^12^

In recent decades, aberrant states of cohesin in human cancers have been discovered in terms of both mutations and expression levels. On the one hand, cohesin is among the most commonly mutated protein complexes in cancer,^13^ and mutations in cohesin are frequent genetic drivers in cancer.^14^ On the other hand, aberrant expression levels of cohesin genes have been observed in many cancer types and are associated with cancer prognosis and metastasis.^15^^,^^16^ To explain the functional impact of aberrant cohesin, early studies attempted to interpret the defective segregation of sister chromatids (i.e., aneuploidy) as the primary phenotypic outcome.^17^ Instead, researchers have recently reached a consensus that aberrant cohesin leads to alterations in chromosome structure and transcription that drive tumorigenesis.^13^ For example, mutations of the cohesin subunit STAG2 in acute myeloid leukemia induce longer chromatin loops and intermixing of compartments;^14^ recurrent cancer mutations in the cohesin subunit SMC1A result in impaired chromatin loops and gene expression;^18^ and reduced expression of the cohesin subunit Rad21 alters intrachromosomal interactions and creates an active transcriptional environment for cancer genes.^16^ Despite these efforts focusing on either mutated cohesin or misexpressed cohesin genes, however, it remains unclear how aberrant cohesin is implicated in human cancers. For example, the presence and role of chromatin-binding abnormalities of cohesin in human cancers are largely unknown.

Given that cohesin needs to bind to chromatin to perform its functions, investigating cancer-specific cohesin binding could be a more direct way to uncover the functional impact of aberrant cohesin. Theoretically, the aberrant binding of cohesin could be attributed to mutations in cohesin structure, dysexpression of cohesin genes, or other environmental factors, as cohesin binding is an epigenetic event (Figure S1A). Here, instead of considering the aberrant state of cohesin itself (e.g., mutation, overexpression), we aim to study the aberrant chromatin-binding events of cohesin between cancer and normal cells (hereafter, aberrant cohesin binding). Of note is a report on cancer-specific CTCF (CCCTC-binding factor) binding in various cancer types.^19^ Although some cohesin colocalizes with CTCF, there is growing evidence that cohesin is different from CTCF.^20^^,^^21^ To date, there have been few comprehensive genome-wide analyses of aberrant cohesin-binding patterns and their functional links to various types of cancer.

To this end, we performed a data-driven analysis based on 550 chromatin immunoprecipitation sequencing (ChIP-seq) datasets, which allowed us to identify the cancer-aberrant binding sites of cohesin (CASs). To study their functional impact, we integrated CASs with transcriptomes (RNA-seq), 3D genomes (Hi-C, ChIA-PET), epigenomes (ReMap,^22^ RoadMap^23^), and clinical information (TCGA,^24^ COSMIC^25^). Our results showed that approximately 1% of the cohesin sites were identified as CASs in a tissue-specific manner. Importantly, CASs were enriched for cancer-dysregulated genes that are functionally and clinically significant. On CASs, we observed alterations in the chromatin compartments, chromatin loops, and *cis*-regulatory modules (CRMs), providing insight into how CASs affect gene expression in cancer cells. Cohesin depletion did affect genes associated with CASs, indicating the active regulatory function of CASs. In addition, we found that CASs were partially associated with DNA methylation but not with somatic mutations. Overall, our integrative data-driven analysis revealed that aberrant cohesin binding is a crucial epigenetic signature that contributes to dysregulated chromatin structure and gene expression in cancer cells.

## Results

### Identification of aberrant cohesin-binding sites in cancer cells

**Figure 1:**
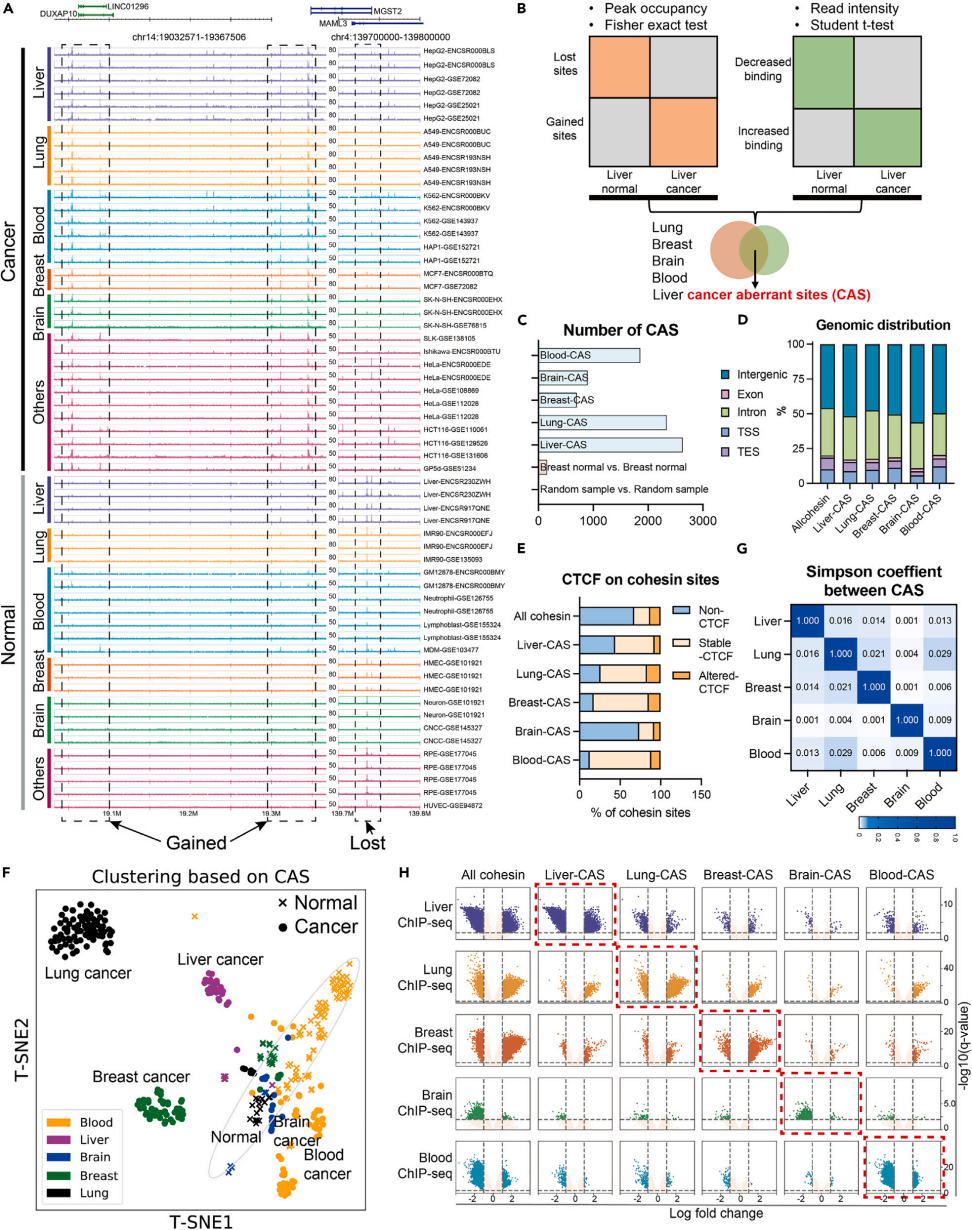
Identification and characteristics of CASs (A) Read distributions of cohesin ChIP-seq in example regions of gained or lost cohesin sites. The numbers on the track represent the scale. The same scale was used for the same tissue type. Dashed rectangles indicate the lost- or gained-cohesin sites when comparing cancer cells to normal cells. (B) Workflow for identifying CASs. We obtained CAS for five tissue types by overlapping the results of peak occupancy and read intensity. (C) The number of CASs identified in the five tissue types. Normal or random samples were used as negative controls. (D) Genomic distribution (RefSeq reference gene annotation) of CASs or other cohesin sites. (E) Changes in CTCF binding from normal to cancer samples at CASs or other cohesin sites. (F) The T-SNE plot shows that cohesin binding in CAS regions generates clustering of ChIP-seq samples. Each dot indicates a ChIP-seq sample, and the color indicates the tissue type. (G) Simpson correlation coefficient between CASs from different tissue types. (H) Volcano plots show the trend of cohesin binding from normal to cancer cells. Each row represents ChIP-seq data for different tissue types. Each column represents different cohesin sites. X axis: log fold change of ChIP-seq read intensity at indicated sites. Y axis: -log10(q-value).

To obtain comprehensive information on cohesin binding, we collected 550 high-quality cohesin ChIP-seq datasets from the GEO^26^ and ENCODE projects^27^ (Table S1). By comparing the 295 cancer cell datasets with the 255 normal cell datasets, we observed that some genomic regions exhibited specific cohesin-binding patterns. For example, Figure 1A shows “gained” sites where cancer samples, but not normal samples, exhibited cohesin binding. In contrast, at the “lost” site, only normal samples exhibited cohesin binding. Notably, high expression of genes near the gained cohesin sites or low expression of genes near the lost cohesin sites indicated poor cancer prognosis (Figures S1B and S1C). In this study, we focused on such CASs that represent different epigenomic states between cancer and normal cells.

To systematically identify CASs, we first pooled the ChIP-seq peaks called from each dataset and defined a “peak occupancy” as the number of ChIP-seq samples exhibiting peaks within a cohesin site.^19^ We identified a total of 748,692 high-confidence cohesin-binding sites with peak occupancy≥2 (Figures S1D and S1E). CASs were then calculated by combining the differential peak occupancy and the differential read intensity between cancer and normal samples (Figure 1B and STAR Methods). Due to data limitations (Table S1), we were able to identify CASs for five tissue types (liver, lung, breast, brain, and blood) for which both cancer and normal samples were available. The number of obtained CASs ranged from 701 to 2,622 (Figure 1C). Approximately 50% of CASs were located in intergenic regions, and the genomic distribution of CASs was not very different from that of other cohesin sites (Figure 1D). Notably, although some cohesin is known to co-act with CTCF, we observed that the majority of CASs (∼90%) were either non-CTCF sites or colocalized with stable CTCF (Figure 1E), which is similar to the proportion of all cohesin sites (88.5%). This result suggested that CASs do not often cooperate with aberrant CTCF binding in cancer cells.

Although CASs were designed to capture the differential cohesin binding between normal and cancer cells for each tissue type (Figures S1F and S1G), the relationship between CASs of different tissue types is unknown. We therefore generated a matrix of ChIP-seq read intensities in the pooled CAS regions. The T-SNE plot in Figure 1F shows that cohesin-binding information at CASs was sufficient to classify the tissue types of cancer samples, while normal samples were clustered into a relatively close group. For comparison, similar clustering could not be achieved by using other cohesin sites (Figure S1H). This suggests that CASs represent signature features that can distinguish cancer samples from normal samples and distinguish one cancer type from others. To directly assess the overlap between CASs from different tissue types, we calculated pairwise Simpson coefficients (Figure 1G). The very low values suggested a high degree of tissue specificity for CASs. In addition, we examined trends in cohesin binding across five tissue types. Figure 1H shows that in the CAS regions of a particular tissue type, cohesin binding did not exhibit much significant change except for the ChIP-seq samples of the corresponding tissue (red rectangle). The histogram in Figure S1I also shows similar trends in peak occupancy. Therefore, changes in cohesin binding at CASs are also tissue specific.

Taken together, these observations suggest that CASs represent epigenomic signatures of cancer cells in a tissue-specific manner.

### CASs are enriched with dysregulated genes in cancer cells

**Figure 2:**
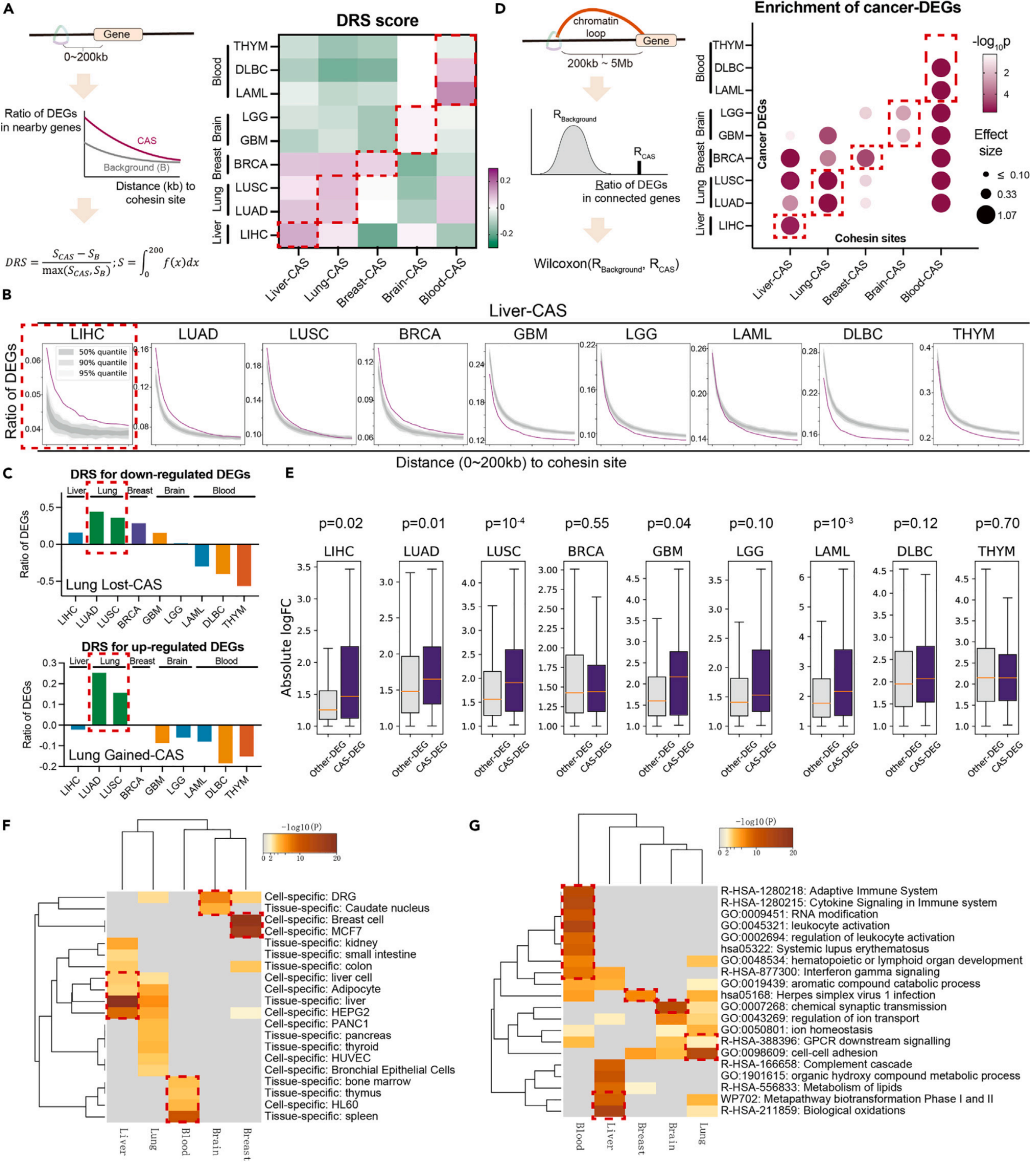
CASs are associated with dramatically dysregulated genes in cancer cells (A) Definition of DEG ratio score (DRS) and DRS results across cancer types. LIHC: Liver hepatocellular carcinoma; LUAD: Lung adenocarcinoma; LUSC: Lung squamous cell carcinoma; BRCA: Breast invasive carcinoma; GBM: Glioblastoma multiforme; LGG: Brain Lower Grade Glioma; LAML: Acute Myeloid Leukemia; DLBC: Lymphoid Neoplasm Diffuse Large B-cell Lymphoma; THYM: Thymoma. (B) Ratio of DEGs against distance from liver-CASs in different cancer types. Pink line: CAS; Gray area: background model with quantiles. The red rectangle shows a higher ratio of liver-DEGs near liver-CASs. Results of other CAS are shown in Figure S2A. (C) DRS scores for gained- or lost-CAS across cancer types. Results of other CAS are shown in Figures S2B and S2C. (D) Definition of the “loop model” and results of DEG enrichment between different types of CAS and different types of DEGs. The p value and effect size were calculated by Wilcoxon test. (E) Comparison of absolute log fold change between CAS-DEGs (DEGs within 5 kb from CASs) and other-DEGs for different cancer types. p values were calculated by the Mann–Whitney U test. (F and G) Enrichment of CAS-related genes in terms of cell types (F) and ontology pathways (G) (GO, KEGG, Reactome).

Considering the widely accepted view that cohesin is a direct regulator of gene expression,^13^ we hypothesized that aberrant cohesin binding at CASs would induce dysregulated genes in cancer cells, i.e., differentially expressed genes (DEGs) between normal and cancer RNA-seq datasets (Table S2). To quantitatively evaluate the enrichment of DEGs near CASs (0–200 kb, “nearby model”), we defined a DEG ratio score (DRS) ranging from −1 to 1 (Figure 2A left, STAR Methods). Positive DRS values indicate that more DEGs are located near the given genomic loci compared to the background model. Figure 2A shows that positive DRS values were frequently observed between CASs of a tissue type and DEGs of the corresponding cancer types (red rectangle), whereas there were some exceptions. Details of the DEG enrichment near CASs are illustrated in Figures 2B and S2A, where genes closer to CASs exhibited higher ratios of cancer DEGs. Furthermore, we did the similar analysis by separating gained- or lost-CAS. Considering that cohesin is present in almost all active enhancers^28^ and helps bring enhancers into proximity to the promoters,^13^ it is reasonable to hypothesize that gained cohesin sites are enriched for upregulated cancer DEGs, while lost cohesin sites are enriched for downregulated cancer DEGs. Indeed, we observed frequent positive DRS values for upregulated genes near gained-CASs and for downregulated genes near lost-CASs (Figures 2C, S2B, and S2C).

On the other hand, cohesin is known to mediate gene expression via long-distance chromatin loops.^29^ We therefore investigated the proportion of DEGs in genes linked to CASs via chromatin loops^30^ (200 kb–5 Mb, “loop model”). By comparing the background ratios, we frequently observed significant enrichment between CASs of a tissue type and DEGs of the corresponding cancer type (Figure 2D). Similar to the nearby model, the loop model was also analyzed for gained- and lost-CAS separately. As revealed in Figures S2D and S2E, upregulated DEGs and downregulated DEGs were likely to be enriched in gained-CASs and lost-CASs, respectively.

Combining the results of the nearby model and loop model, we suggest that CASs are epigenomic signatures that are highly associated with the presence of transcriptomic abnormalities in cancer cells.

### CAS-related DEGs are functionally and clinically important genes

It is worth noting that not all cancer DEGs were associated with CASs (Figure S3A). To acquire distinctive features of CAS-DEGs, we first analyzed gene expression levels. Compared to other-DEGs, CAS-DEGs did not show consistently higher or lower levels of gene expression across cancer types (Figure S3B). We then analyzed the degree of change in gene expression. Importantly, in most cancer types, CASs-DEGs always had larger absolute values of log fold change than other-DEGs (Figure 2E), and this trend diminished as DEGs were distant from CASs (Figures S3C and S3D). This suggests that CASs are associated with the most markedly dysregulated genes in cancer cells.

We also compared CAS-DEGs from different tissue types. Not surprisingly, there was little overlap between CAS-DEGs from different tissue types (Figure S3E). Figure 2F shows that CAS-DEGs were enriched for the pattern genes^31^ of the corresponding tissue types (red rectangle); for example, liver CAS-DEGs were enriched for liver tissue-/cell-specific genes. Functional enrichment^32^ (Figures 2G and S3F) showed that CAS-DEGs were enriched in pathways that are important for the respective cancer types (red rectangle), e.g., “biological oxidation” for liver cancer.^33^ We further conducted functional enrichment analyses for gained- and lost-CAS separately, where we also observed cancer type-specific pathways (red rectangles in Figure S3G). These observations illustrate the specificity and functional importance of CAS-DEGs.

**Figure 3:**
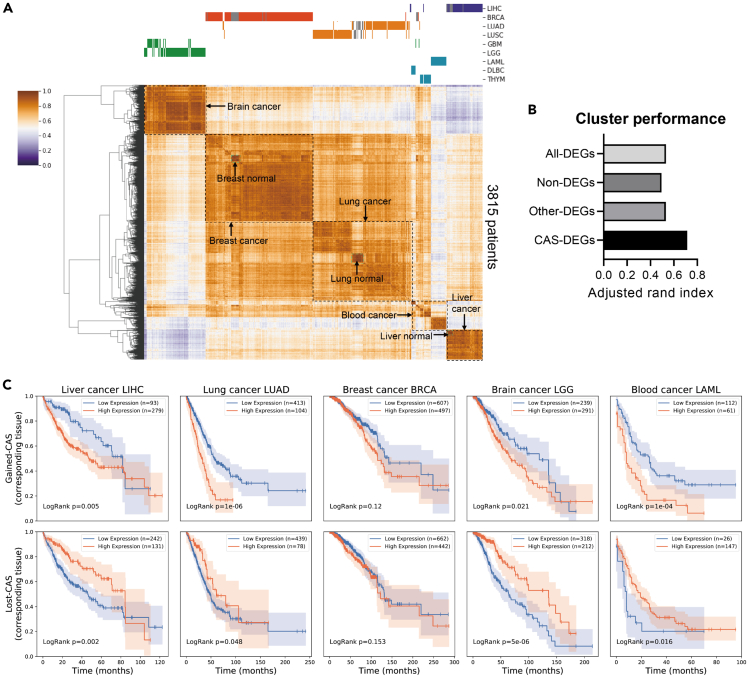
CAS-related DEGs are clinically important genes (A) Clustering of 3815 patient samples based on gene expression levels of CAS-DEGs. Cancer types are labeled at the top, and normal samples are labeled as gray bars. (B) The adjusted rand index obtained for different gene lists. The same number of genes as CAS-DEGs were sampled from non-DEGs or other-DEGs to conduct the analysis. All-DEGs pooled DEGs from all cancer types. (C) Kaplan‒Meier survival analysis for gained-CAS DEGs (upper) or lost-CAS DEGs (bottom). p values were obtained by the log rank test. Blue lines represent the low expression group, while orange lines represent the high expression group.

Given that CAS-DEGs are tissue-specific genes that vary dramatically from normal to cancer, we hypothesized that CAS-DEGs would be informative for classifying patient samples. Figure 3A shows that the expression levels of CAS-DEGs could successfully classify 3815 TCGA patient samples into their respective groups, including different cancer groups and the related normal groups. In contrast, classification based on other-DEGs, all-DEGs, or non-DEGs generated poorer clustering, as evidenced by more breaks at the top of the heatmap (Figure S3H). To quantitatively evaluate cluster performance, we used the adjusted rand index and observed that CASs-DEGs provided a superior score than other-DEGs, all-DEGs, or non-DEGs (Figure 3B). Therefore, the identification of CASs can help find signature genes in clinical samples of various types of cancer, providing a research basis for cancer diagnosis and therapeutic treatment.

Another aspect of clinical importance was obtained from the survival analysis. Because CASs-DEGs are a list of genes rather than a single gene, we used the GSVA algorithm^34^ to stratify patients into two groups. Kaplan‒Meier analysis showed that high expression of gained-CASs-DEGs and low expression of lost-CASs-DEGs denoted poor prognosis (Figure 3C, 8 out of 10 panels exhibited significant p values <0.05, log rank test). The pathway enrichment in Figures 2G and S3G might contribute to the poor prognosis outcomes observed in Figure 3C. For example, the estrogen signaling pathway observed for gained-CAS, when enhanced, promotes the development and progression of breast cancer^35^^,^^36^; the immune response pathways observed for lost-CAS, when suppressed, are associated with poor survival rates in patients with blood cancer.^37^^,^^38^ Combining the correlation between CASs and up- and downregulated genes, we speculate that gained-CASs-DEGs are more likely to be oncogenes, while lost-CASs-DEGs are more likely to be tumor suppressors. Indeed, we examined the cancer gene census^25^ and observed a higher ratio of oncogenes for gained-CAS-DEGs and a higher ratio of suppressor genes for lost-CAS-DEGs (Figure S3I), indicating the potential role of CAS for cancer development and progression.

Collectively, CASs are associated with functionally and clinically important genes that vary dramatically from normal tissue to cancer cells.

### CASs are related to altered chromatin compartments

Cohesin is thought to regulate gene expression by directing the organization of 3D genomes at different layers.^13^ To understand how CASs are linked to dysregulated genes in cancer cells, we collected and analyzed 3D genomic datasets (Table S3) for liver, lung, breast, and blood samples.

**Figure 4:**
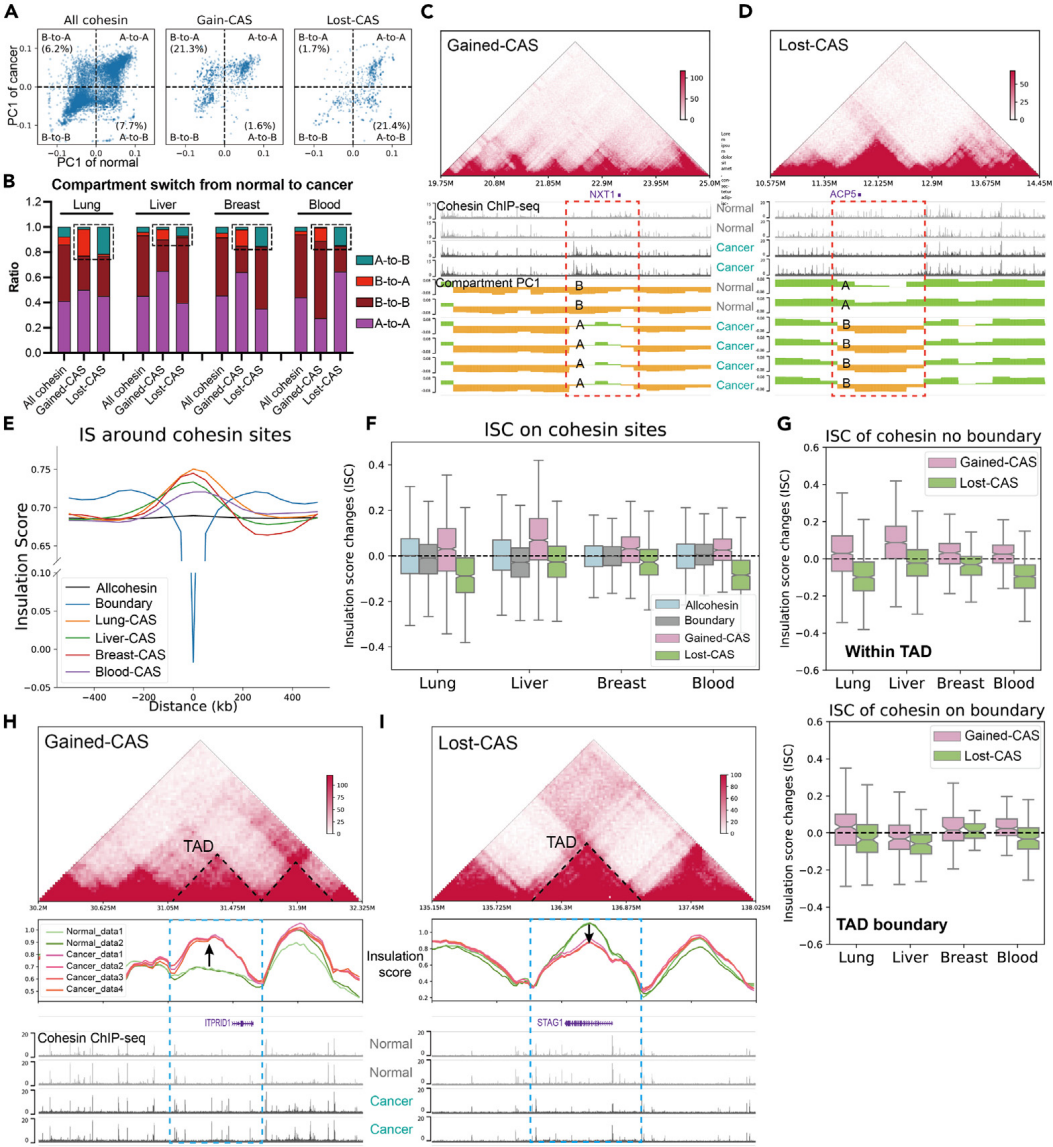
Chromatin compartments and TADs on CASs (A) Scatterplot of compartment PC1 in lung normal (x axis) and lung cancer (y axis) samples for all cohesin, gained-CAS or lost-CAS. Each dot represents a cohesin site. (B) Proportions of compartment switches from normal to cancer for different cohesin sites. A locus with $E_{cancer}\times E_{normal}<0$ and ${|E}_{cancer}-E_{normal}|>0.05$ was defined as a compartment switch, where E is the average PC1 values of multiple Hi-C samples. Dashed rectangles suggest the elevated B-to-A switch at gained-CAS and the elevated A-to-B switch at lost-CAS. (C and D) Example regions show Hi-C contacts, compartment PC1, and cohesin binding near the gained-CASs (C), or the lost-CASs (D) in lung normal cells IMR-90 and lung cancer cells A-549. Green indicates compartment A and yellow indicates compartment B. Expression levels of related genes are shown in Figure S4C. (E)Aggregation plot of IS near (±500 kb) different cohesin sites. “Boundary” represents cohesin sites located on the TAD boundary. (F)Boxplot of ISC at all cohesin, boundary cohesin, gained-CAS and lost-CAS. The horizontal line indicates 0. (G)Boxplot of ISC at CAS regions within TADs (upper) or on TAD boundaries (bottom). (H and I) Example regions show Hi-C contacts, insulation scores, and cohesin binding near the gained-CASs (H), or the lost-CASs (I) in lung samples. Expression levels of related genes are shown in Figure S4F.

We first examined the chromatin compartment, the megabase structure that partitions human genomes into active compartment A and inactive compartment B.^13^^,^^39^ Figure S4A illustrates that approximately half of the CASs were in compartment A, which is similar to the proportion of all cohesin sites. We then studied the change in compartments from normal to cancer samples, i.e. compartment switches^40^ (Figures 4A, 4B and S4B). A locus is considered a compartment switch if its PC1 value has opposite signs in cancer and normal cells and the absolute difference between these values is greater than 0.05. Across the four tissue types available, gained-CASs always showed higher proportions of B-to-A switches but lower proportions of A-to-B switches compared to all cohesin sites, while lost-CASs always showed lower B-to-A proportions but higher A-to-B proportions. For example, in lung cancer, all cohesin sites exhibited 6.2% B-to-A and 7.7% A-to-B switches; gained-CASs exhibited 21.3% B-to-A and 1.6% A-to-B switches; lost-CASs exhibited 1.7% B-to-A and 21.4% A-to-B switches (Figure 4A). Representative regions further illustrated the B-to-A compartment switches and upregulated genes near gained-CASs, and the A-to-B compartment switches and the downregulated genes near lost-CASs (Figures 4C, 4D, and S4C).

Considering the correspondence between compartment switch and gene expression,^40^ the above results could partly explain how CASs are correlated with cancer-dysregulated genes. However, only 10%–20% of CASs were involved in compartment switches. We therefore studied the deeper layer of chromosome structure.

### CASs are related to the alteration of intra-TAD interactions

Cohesin is known to organize TADs,^41^ the functional units that regulate gene expression by restricting CRMs.^42^ Our Hi-C analysis revealed that ∼10% of CASs are located at TAD boundaries, which is similar to the proportion of all cohesin sites (Figure S4D). For quantitative comparison, we used the insulation score (IS), which measures interactions passing across each genomic locus.^40^ IS has local minima at highly insulated regions, i.e., TAD boundaries. Aggregation plots of IS showed that all cohesin sites appeared as a flattened line, whereas the positive control (cohesin sites on boundaries) exhibited a sharp negative peak (Figure 4E). In contrast, we observed peak enrichment at CASs but local minima away from CASs. Figure S4E also shows that CASs had higher IS values than boundary cohesin or all cohesin. These results suggest that CASs tend to reside inside TADs rather than at TAD boundaries.

To compare cancer and normal samples, we examined insulation score changes (ISC).^40^ The ISC, which quantifies changes in local chromosomal contacts, was calculated as the difference between the IS value of cancer samples and the IS value of normal samples. Disruption of TAD boundaries has been found in cancer cells.^43^ Considering the functional roles of cohesin in TAD formation,^41^ we hypothesize, for example, that loss of cohesin binding would lead to loss of boundaries, resulting in greater IS values (i.e., positive ISC). Unexpectedly, we observed negative ISC on lost-CASs, but positive ISC on gained-CASs (Figure 4F), suggesting there is a decrease in local chromosomal contacts around lost-CAS and an increase in local chromosomal contacts around gained-CAS. Therefore, the ISC results at CASs cannot be explained by the disruption of boundaries. Instead, since CASs tend to be present within TADs and ISC also represents changes in local chromosomal contacts, the ISC observations could suggest that CASs are more relevant to changes in local contacts within TADs rather than boundary disruptions. To test this conclusion, we further studied CASs on boundaries or within TADs. Figure 4G shows that there was a greater extent of changes in ISC within TAD than on TAD boundaries, indicating that CASs are more likely to contribute to the disruption of intra-TAD local contacts rather than boundaries. Representative genomic regions showed elevated IS and upregulated genes near gained-CASs and decreased IS and downregulated genes near lost-CASs (Figures 4H, 4I, and S4F), while IS on boundaries did not change much. Interestingly, lost-CASs were associated with downregulation of the cohesin subunit gene STAG1 (Figures 4I and S4F), indicating a positive feedback loop that further disturbs the cohesin states in cancer cells.

**Figure 5:**
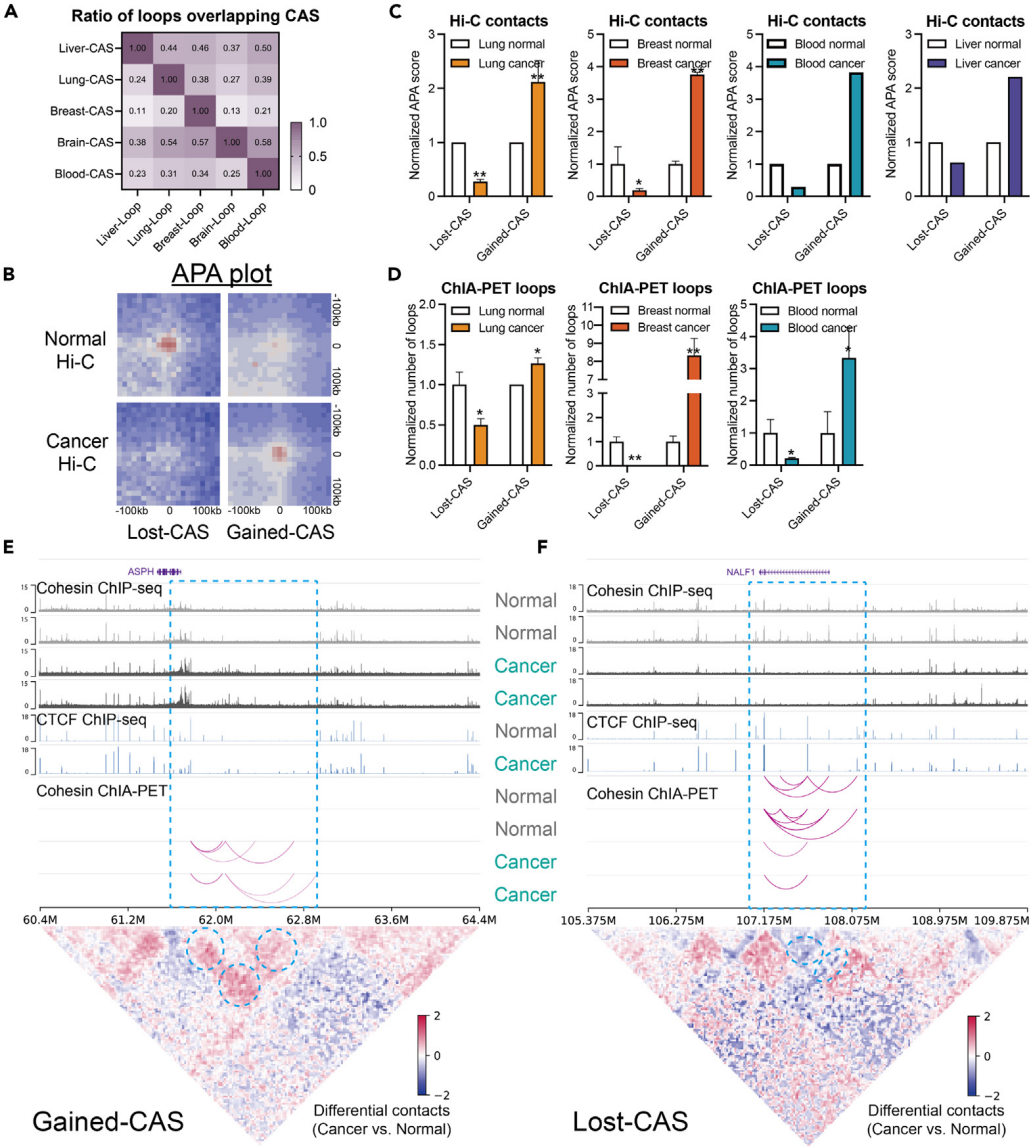
Altered chromatin loops at CASs (A) Ratios of chromatin loops overlapping with CASs across tissue types. The ratio is normalized by the overlap of CASs and loops in the same tissue type. (B) The APA plot shows the decreased contacts around (±100 kb) lost-CASs, and increased contacts around gained-CASs. (C) Normalized APA scores for lost- and gained-CAS in four available tissue types. Normal samples were normalized as 1. ∗: p < 0.05; ∗∗: p < 0.01; One-sided t-test. (D) Number of chromatin loops detected by ChIA-PET in normal or cancer cells. Normal samples were normalized as 1. ∗: p < 0.05; ∗∗: p < 0.01; One-sided t-test. (E and F) Example regions show cohesin binding, CTCF binding, ChIA-PET loops, differential Hi-C contacts near the gained-CASs (E), or the lost-CASs (F) in lung normal samples and lung cancer samples. Blue circles indicate increased or decreased Hi-C contacts.

To further study the disruption of intra-TAD interactions, we then focused on chromatin loops, the cohesin-mediated structures that bring distal elements into close physical proximity.^44^ The length of loops anchored at CASs did not differ from that of all cohesin sites (Figure S4G). The overlap between chromatin loops and CASs suggested that the CAS-anchored loops were also tissue specific (Figure 5A). To measure changes in chromatin loops, we performed aggregate peak analysis (APA) on the Hi-C samples of normal and cancer cells. Figure 5B shows that chromatin loops centered on lost-CASs were weakened, whereas those on gained-CASs were strengthened. Quantification by normalized APA score revealed consistent trends across the four tissue types (Figure 5C). ChIA-PET analysis also revealed that gained-CASs and lost-CASs were related to the gain and loss of cohesin loops, respectively (Figure 5D). For comparison, all cohesin sites did not exhibit similar differences (Figures S4H and S4I), suggesting that CASs are specifically related to modified chromatin interactions in cancer cells. In representative regions (Figures 5E, 5F, and S4J), gained-CASs showed established ChIA-PET cohesin loops, strengthened Hi-C contacts, and upregulated genes. In contrast, lost-CASs exhibited loss of ChIA-PET loops, decreased Hi-C contacts, and downregulated genes. In particular, CTCF binding did not change in these regions (Figures 5E and 5F), indicating that CASs could influence chromatin loops independent of CTCF.

Together, we suggest that CASs are highly associated with alterations in chromatin loops inside TADs, but less likely related to boundary disruptions.

### CASs are required for gene dysregulation by altering *cis*-regulatory modules in cancer cells

**Figure 6:**
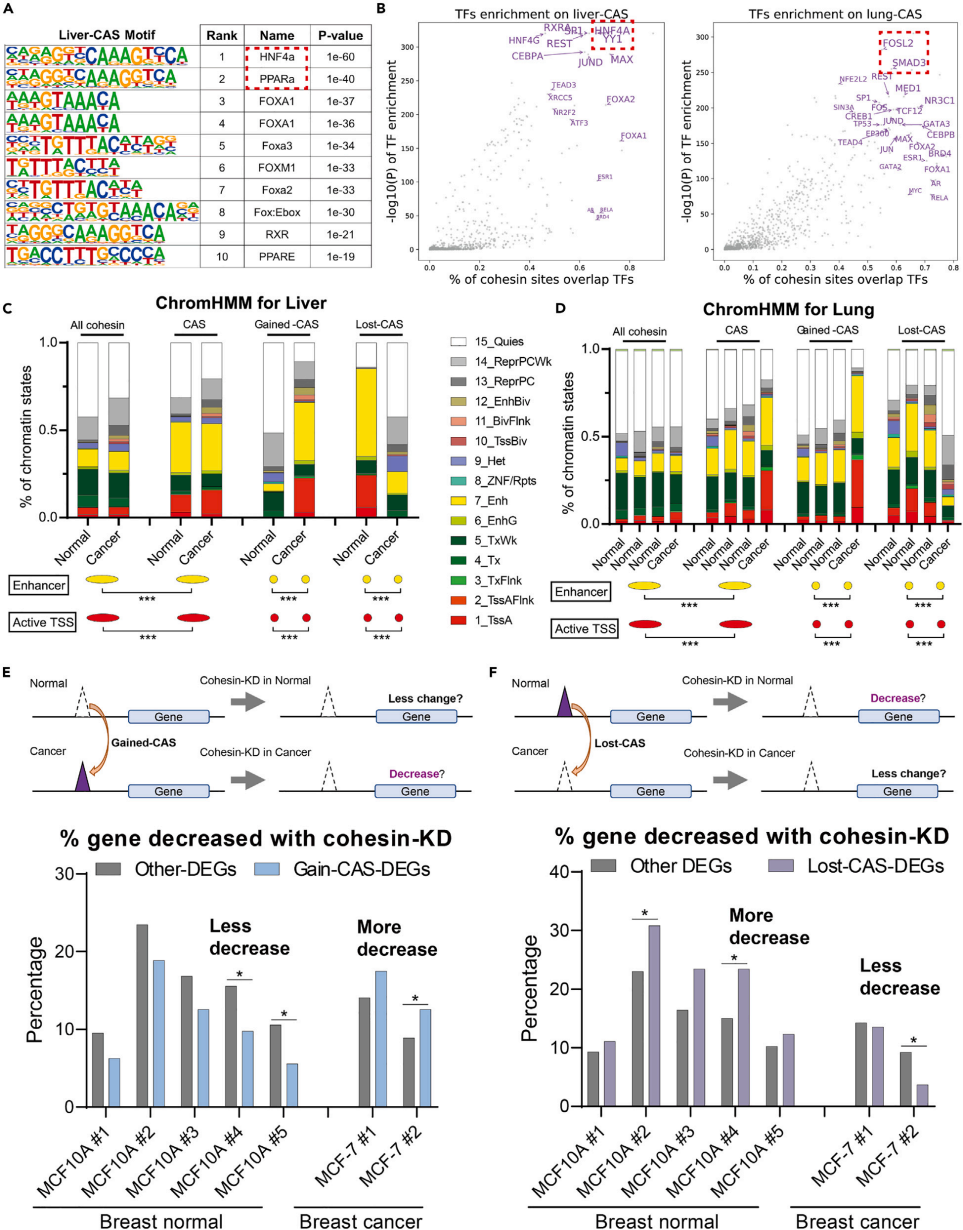
*Cis*-regulatory modules at CASs (A) Top 10 binding motifs for liver-CASs. Red rectangles indicate TFs known to be important for liver cancer. (B) Enrichment of TFs at liver-CASs and lung-CAS. The x axis indicates what percentage of CAS sites overlap with TF, while the y axis is the -log10 p value obtained by comparison between the selected CASs and all cohesin sites. Each dot represents a type of TF. The TFs in the upper right are indicated to be highly enriched at given cohesin sites. (C) Proportions of liver normal and liver cancer chromatin states at liver-CASs. ∗∗∗: p < 0.001; Fisher’s exact test. (D) Proportions of lung normal and lung cancer chromatin states at lung-CASs. ∗∗∗: p < 0.001; Fisher’s exact test. (E–F) Hypothesis and ratio of decreased genes after cohesin depletion in the indicated groups. ∗: p < 0.05; Fisher’s exact test.

Chromatin interactions within TADs are the structural basis for forming CRMs that control gene expression.^5^ Given the altered intra-TAD interactions near CASs, we subsequently studied CRMs. We first examined the enrichment of transcription factors (TFs) by motif analysis. The results showed that CASs of one tissue type were enriched with significant TFs for the corresponding cancer type (Figures 6A and S5A, red rectangle). For example, liver-CASs were enriched with motifs of HNF4a and PPARa, which are vital TFs for the development of liver cancer.^45^ In addition to computational motif prediction from DNA sequences, we also analyzed the ChIP-seq peaks of 1136 TFs.^22^ By considering the proportion of overlap and statistical significance (Methods), we found that CASs of one tissue type were usually enriched with important TFs of the corresponding cancer type (Figures 6B and S5B, red rectangle). These results suggested that CASs were associated with tissue-specific CRMs that bind many important TFs for cancer.

To study the changes in CRMs from normal to cancer cells, we then analyzed the chromatin state, which integrates five histone markers to segment all genomes into 15 types of elements.^23^ As shown in Figures 6C, 6D, and S5C, we observed significantly higher ratios of active enhancers and promoters on CASs than on all cohesin sites. Importantly, from normal to cancer cells, the ratio of active enhancers and promoters increased significantly at gained-CASs, but decreased at lost-CASs (data available for lung, liver, and blood). This suggested that CASs are particularly associated with altered enhancer-to-promoter machineries in cancer cells.

To verify that aberrant cohesin binding does have a function at CASs, we analyzed RNA-seq data with cohesin depletion (Table S4). Since we assumed that CAS-related dysregulated genes result from aberrant cohesin binding, we expected that cohesin depletion would have greater effects on CAS-DEGs than on other-DEGs or non-DEGs. Indeed, CASs-DEG exhibited a higher proportion of altered genes in MCF-7 cells, and these genes were enriched in breast cancer-specific pathways (Figures S5D and S5E). This finding supported the idea that changes in cohesin binding at CASs regulate important genes in cancer cells.

We further investigated cohesin depletion for gained- or lost-CASs separately. By definition, cohesin depletion should affect gained-CASs more in cancer cells, but less in normal cells (Figure 6E). Combined with the enrichment of upregulated genes near gained-CASs, we assumed that cohesin depletion would downregulate gained-CASs-related genes in cancer cells but have less impact in normal cells. Indeed, in breast cancer cells, cohesin depletion induced more decreases in gained-CAS-DEGs than in other-DEGs, while in breast normal cell there were fewer decreases in gained-CAS-DEGs (Figure 6E, bottom). This observation was consistent across seven public datasets, three of which were statistically significant. In contrast, we expected an opposite phenomenon for lost-CASs. That is, in normal cells, cohesin depletion would decrease the expression levels of lost-CASs-related DEGs, whereas in cancer cells cohesin depletion has less effect because cohesin binding at lost-CASs would not be further altered (Figure 6F). As expected, we observed more decreased genes in breast normal cells but fewer decreased genes in breast cancer cells (Figure 6F). These results suggest that at least some aberrant cohesin binding in CAS regions is required for cancer-dysregulated genes.

Taken together, we conclude that CASs could induce dysregulation of gene expression by introducing abnormalities in the enhancer-to-promoter CRMs of cancer cells.

### CASs are partially related to differential DNA methylation but not to somatic mutations

Cohesin has been shown to preferentially bind to hypomethylated DNA regions in Cornelia de Lange disease.^46^ In cancer cells, CTCF binding is thought to be negatively correlated with DNA methylation levels.^6^^,^^19^ Therefore, we examined whether there was a negative correlation between cohesin binding and DNA methylation at CASs. As shown in Figures S6A and S6B, lost-CASs did exhibit a peak-like enrichment for increased DNA methylation, which was significantly higher than that of random cohesin sites. Interestingly, however, gained-CASs showed neither a higher proportion of decreased DNA methylation nor peak-like aggregations (Figures S6C and S6D). One possibility is that hypermethylation rejects cohesin binding, thereby introducing lost-CASs, whereas hypomethylation does not recruit new cohesin binding and therefore has no association with gained-CASs (Figure S6E). Combined with the fact that DNA methylation at CAS regions could hardly classify patient samples (Figure S6F), we conclude that CASs are partially related to differential DNA methylation.

Lastly, we studied somatic mutations in CAS regions, as cohesin-binding sites are known to be frequently mutated in cancer.^28^ Although we did observe an enrichment of mutations near cohesin sites (Figure S6G), we did not observe more coding mutations or noncoding variants in CAS regions than in other cohesin sites (Figure S6H). In contrast, we observed enrichment of somatic mutations at cohesin sites that were conserved between normal and cancer cells. These results suggest that differential binding events of cohesin are not necessarily associated with more or less somatic mutations.

## Discussion

Mutations in cohesin are suggested to be the major cause of human cancer.^13^^,^^14^^,^^15^^,^^17^^,^^18^ Other studies have focused on the unusual expression levels of cohesin genes in different types of cancer.^15^^,^^16^^,^^47^^,^^48^^,^^49^^,^^50^^,^^51^ However, the specific mechanisms by which aberrant states of cohesin contribute to cancer remain unclear.^13^ In contrast to previous studies that mainly discussed abnormalities in cohesin per se, we present the first comprehensive identification of aberrant cohesin-binding sites (i.e., CASs) across cancer types. Through integrated analysis of large-scale public datasets, we systematically investigated whether and how CASs are associated with dysregulated genes in cancer cells. Especially, our observations were consistent among cancer types, types of evidence (e.g., gained-CASs were related to upregulated genes, activated compartment, and more active CRMs), and assay types (e.g., ChIA-PET loops and Hi-C loops), providing convincing results for the presence and impact of aberrant cohesin binding in cancer cells.

Although our initial glimpse observed a consistent gain or loss of cohesin binding across tissue types (Figure 1A), genome-wide surveys revealed that CASs were highly tissue specific. Given that tissue-specific cohesin is more likely to be independent of CTCF^20^^,^^21^ and our results in Figure 1G, we suggest that CASs are unlikely to be associated with changes of CTCF. Importantly, despite CASs being obtained from ChIP-seq data of cell lines, we observed strong correlations between CASs and patient data (i.e., gene expression, survival), indicating that CASs are widely existing phenomena across cell lines and patient cells. Notably, not all cancer-dysexpressed genes were related to CASs. CASs tend to be associated with the most altered genes that are functionally and clinically significant. Therefore, CASs represent a cluster of epigenetic regulatory elements that contribute to transcriptional dysregulation in cancer cells.

Cohesin is known to mediate multiple layers of chromatin structure.^5^ Around CASs, we observed alterations in chromatin compartments and loops. Although alterations in TAD boundaries have been reported in human cancers,^8^^,^^9^^,^^10^^,^^43^ CASs were not enriched for altered TAD boundaries, but were associated with remodeling of tissue-specific CRMs within TADs. One explanation is that TAD boundaries are highly conserved; hence, the actual proportion of disrupted boundaries is fairly low in cancer cells.^52^ In addition, disruption of boundaries has been reported to require abnormal CTCF binding.^52^ Considering that CASs did not colocalize with altered CTCF, it is reasonable that CASs do not affect boundaries.

Somatic mutations in cohesin-CTCF-binding sites have been reported to occur frequently in cancer.^7^^,^^28^ Whereas enriched mutations were indeed observed near cohesin sites, CASs did not exhibit different mutation levels compared to other cohesin sites. This suggests that aberrant cohesin-binding events can rarely be attributed to somatic mutations, which is similar to the findings for cancer-specific CTCF.^19^ Conversely, we observed more mutations for conserved cohesin sites, further supporting that somatic mutations are not necessarily associated with aberrant binding events of cohesin.

Overall, we conducted an integrative computational analysis on large-scale multiomics data. Our comprehensive investigations identified aberrant cohesin binding as a significant epigenetic signature in cancer cells. Our work provides new insight into cancer epigenomics and cohesin cancer biology.

### Limitations of the study

In this study, although we tried to find the most accordant phenomena across tissue types and data types, our data-driven analysis was still limited by data availability. After all, CAS regions were derived from public ChIP-seq data for normal or cancer cell lines, by which we could roughly classify five tissue types. The identification process might be affected by noise factors, such as different antibody, different cell lines in the same tissue type, experimental protocols, and other technical variations. We were unable to analyze more details, such as cancer subtypes. Ideally, aberrant cohesin-binding data from patient samples would provide more direct clinical insights into CASs. On the other hand, CASs account for only a restricted proportion (∼1%) of cohesin sites. To obtain high confidence and more specific regions for CASs, some other worthy cohesin sites might be missed. Given that cohesin is a direct regulator of gene expression,^13^ aberrant states of cohesin may have a more general influence on gene dysregulation in cancer cells. In addition, although we have proposed associations between CASs and cancer-dysexpressed genes, the causal relationship is still not very clear. Because cohesin can regulate gene expression through multiple functional mechanisms, it is not straightforward to conclude the role of CAS as a single theory. Future efforts ought to focus on the experimental validation of how CASs disrupt gene regulatory machinery. For example, although we have used cohesin knockdown data to analyze the function of CAS, typical knockdown experiments can also affect other cohesin-binding sites. It would be better to use selective methods (e.g., CRISPR-Cas9) that can specifically perturb cohesin binding on the CAS regions.

## STAR★Methods

### Key resources table

**Table:** 

REAGENT or RESOURCE	SOURCE	IDENTIFIER
**Deposited data**		

ENCODE	Luo et al., 2020^27^	https://www.encodeproject.org/
GEO	Barrett et al., 2013^26^	https://www.ncbi.nlm.nih.gov/geo/
TCGA	Cancer Genome Atlas Research, 2013^24^	https://www.cancer.gov/ccg/research/genome-sequencing/tcga
UCSC Xena	Goldman et al., 2020^53^	https://xena.ucsc.edu/
CohesinDB	Wang et al., 2022^30^	https://cohesindb.iqb.u-tokyo.ac.jp/
ReMap	Hammal et al., 2022^22^	https://remap2022.univ-amu.fr/
Roadmap	Roadmap Epigenomics Consortium, 2015^23^	https://egg2.wustl.edu/roadmap/web_portal/
COSMIC	Tate et al., 2019^25^	https://cancer.sanger.ac.uk/cosmic

**Software and algorithms**		

MACS2	Zhang et al., 2008^55^	https://pypi.org/project/MACS2/
DROMPAplus	Nakato et al., 2021^56^	https://drompaplus.readthedocs.io/en/latest/
Juicer and Juicertools	Durand et al., 2016^60^	https://github.com/aidenlab/juicer
Metascape	Zhou et al., 2019^32^	https://metascape.org/
WashU Epigenome Browser	Li et al., 2022^63^	https://epigenomegateway.wustl.edu/
Mango	Phanstiel et al., 2015^59^	https://github.com/dphansti/mango
STAR	Dobin et al., 2013^57^	https://github.com/alexdobin/STAR
Bowtie2	Langmead et al., 2012^54^	https://bowtie-bio.sourceforge.net/bowtie2/index.shtml
HiC1Dmetrics	Wang et al., 2022^40^	https://h1d.readthedocs.io/en/latest/

### Resource availability

#### Lead contact

Further information and requests for resources and reagents should be directed to and will be fulfilled by the lead contact, Ryuichiro Nakato (rnakato@iqb.u-tokyo.ac.jp).

#### Materials availability

This study did not generate new unique reagents.

### Method details

#### Collection of multiomics data

Raw sequencing data for ChIP-seq, RNA-seq, ChIA-PET and Hi-C data were collected from the NCBI GEO database^26^ and ENCODE project.^27^ Table S1 summarizes the 550 cohesin ChIP-seq datasets used in this study, examined by normalized strand coefficient > 1.2 and peak numbers > 1,000. Table S3 summarizes the information for 45 Hi-C and 14 ChIA-PET datasets. Tables S2 and S4 summarize the 3,815 RNA-seq datasets from UCSC Xena^53^ and 19 RNA-seq datasets with cohesin depletion from the GEO database. DNA methylation and survival data for patient samples were obtained from the TCGA database.^24^ The chromatin loops for analyzing DEG enrichment (‘loop model’) were obtained from CohesinDB.^30^ Transcription factor binding information was obtained from the ReMap database.^22^ Chromatin state annotations were obtained from the Roadmap project.^23^

#### Processing of NGS data

For ChIP-seq data, Bowtie2^54^ was used for alignment, MACS2^55^ was used for peak calling, and DROMPAplus^56^ was used for normalizing reads and generating bigwig files. ChIP-seq reads were visualized by DROMPAplus. For RNA-seq data, reads were mapped using STAR,^57^ gene expression levels were determined using RSEM, and differentially expressed genes were identified using edgeR.^58^ For ChIA-PET data, the MANGO pipeline^59^ was used to extract cohesin-mediated chromatin loops. For Hi-C data, Juicer^60^ was used to generate the contact matrix, and HiCCUPS^60^ was used to detect chromatin loops. All NGS data were aligned to human genome build hg38.

#### Identification of cohesin binding sites and CASs

To generate universal cohesin binding sites with high confidence, we pooled peaks that were found in more than two ChIP-seq datasets. After excluding chromosomes Y and M, we obtained a total of 748,692 cohesin sites. Cancer aberrant cohesin sites (CASs) were then identified based on both peak occupancy and binding levels of cohesin (Figure 1B). On the one hand, for each cohesin site, the binomial peak occupancy was defined as $PO=N_{observed}/N_{all}$ and changes of peak occupancy was $POC={PO}_{cancer}-{PO}_{normal}$, where N is the number of datasets. Statistical comparisons of peak occupancy were conducted using Fisher’s exact test, with multiple testing correction using Benjamini‒Hochberg (BH) adjustment. Cohesin sites with significant changes in peak occupancy were then extracted according to |POC|>0.5 and adjusted P value <0.01. On the other hand, differential cohesin binding levels were calculated by comparing the logarithm read intensity between cancer and normal samples. Cohesin sites with significant changes in binding levels were extracted by |logFC|>1 and adjusted P value <0.01 (t test, B-H adjustment). Thereafter, CASs were obtained by overlapping the results from peak occupancy and binding levels. The tissue specificity of CASs was quantified by the Simpson index, which measures the overlap of peak sets X and Y as:

$$Simpson\left(X,Y\right)=\frac{\left|X\cap Y\right|}{\min\left(\left|X\right|,\left|Y\right|\right)}$$

#### DEGs enrichment by nearby model and loop model

In the ‘nearby model’, we focused on genes related to nearby cohesin binding (Figure 2A). We first calculated the ratio of DEGs that were 0∼200 kb away from the selected cohesin sites (i.e., CASs), yielding a curve representing the ratio of DEGs as a function (f) of genomic distance. The background model (B) was obtained by randomly choosing all cohesin sites 500 times. To evaluate the enrichment of DEGs compared to the background, we defined the DEG ratio score (DRS) as:

$$DRS=\frac{S_{CAS}-S_{B}}{\max\left(S_{CAS},S_{B}\right)};where\;S=\int_{0}^{200kb}f\left(x\right)dx$$

DRS is a score ranging from -1 to 1, where DRS > 0 represents positive enrichment of DEGs. In the ‘loop model’, we focused on genes related to distal cohesin binding through long-distance chromatin loops (Figure 2D). Enrichment of DEGs was then calculated by comparing the DEG ratios of CASs and randomized cohesin sites:

$$Enrichment\;of\;DEGs=Wilcoxon\left(R_{CAS},R_{Background}\right)\text{,}$$

where R is the ratio of DEGs connected to cohesin via chromatin loops. Differentially expressed genes for each cancer type were calculated by GEPIA2^61^ with |log fold change|>1 and false discovery rate FDR < 0.01.

#### Analysis of CAS-related genes

Gene pathway enrichment was performed by Metascape^32^ with an adjusted p value cutoff of 0.01. To obtain distinctive profiles of CASs, cancer DEGs were divided into CAS-DEGs (DEGs near CASs) and other DEGs. Gene expression levels and log fold changes were then compared between CAS-DEGs and other-DEGs. Clustering of the 3815 patient samples was obtained by pairwise correlation followed by hierarchical clustering. We used the adjusted rank index (ARI) to quantify the clustering performance. Specifically, suppose $X_{i}=\left\{X_{1},X_{2},..,X_{9}\right\}$ represents the true label of a cancer type and $Y_{i}=\left\{Y_{1},Y_{2},..,Y_{9}\right\}$ represents the predicted label of a cluster from k-means. Suppose $n_{ij}$ denotes the number of shared patient samples between labels $X_{i}$ and $Y_{i}$; then,

$$ARI=\frac{\sum_{ij}\left(\begin{array}{l} n_{ij}\\ 2 \end{array}\right)-\left[\sum_{i}\left(\begin{array}{l} a_{i}\\ 2 \end{array}\right)\sum_{j}\left(\begin{array}{l} b_{j}\\ 2 \end{array}\right)\right]/\left(\begin{array}{l} n_{all}\\ 2 \end{array}\right)}{\frac{1}{2}\left[\sum_{i}\left(\begin{array}{l} a_{i}\\ 2 \end{array}\right)+\sum_{j}\left(\begin{array}{l} b_{j}\\ 2 \end{array}\right)\right]-\frac{\left[\sum_{i}\left(\begin{array}{l} a_{i}\\ 2 \end{array}\right)\sum_{j}\left(\begin{array}{l} b_{j}\\ 2 \end{array}\right)\right]}{\left(\begin{array}{l} n_{all}\\ 2 \end{array}\right)}}\text{,}$$

where $a_{i}=\sum_{j=1}^{9}n_{ij}$, $b_{j}=\sum_{i=1}^{9}n_{ij}$, and $n_{all}=3815$. Kaplan‒Meier survival analysis of a single gene was performed by GEPIA2.^61^ To conduct survival analysis for gene sets, the GSVA algorithm^34^ was applied to stratify patients into two groups, and the log-rank test was used to determine significance.

#### Analysis of the 3D genome

All Hi-C contact matrices were normalized by the VC_SQRT method. One-dimensional metrics, including compartment PC1, insulation score (IS) and insulation score change (ISC), were calculated by HiC1Dmetrics^40^ at a resolution of 50 kb. A locus with $E_{cancer}\times E_{normal}<0$ and ${|E}_{cancer}-E_{normal}|>0.05$ was defined as a compartment switch, where E is the value of PC1. TAD boundaries and aggregate peak analysis (APA) scores^62^ were identified by Juicertools.^60^ Visualization of example regions was made by the WashU epigenome browser.^63^ After scaling by the total number of reads, differential Hi-C contact matrices were generated by comparing cancer with normal samples.

#### Analysis of cis-regulatory modules

Motifs for CASs were analyzed using HOMER with default parameters. The top 10 motifs with the lowest P values except for CTCF are shown. Chromatin binding of a total of 1136 transcription factors from ReMap was used to calculate the proportion of cohesin sites that overlapped with TFs. The enrichment of TF binding was obtained by comparing CASs with all cohesin sites (Fisher’s exact test). Chromatin states based on five chromatin markers (H3K4me3, H3K4me1, H3K36me3, H3K27me3, H3K9me3) were used to segment the whole genome into 15 types at 200 bp resolution. The Roadmap samples used in this study included liver (E066, E118), lung (E096, E128, E088, E114), breast (E119, E028), brain (E81, E82), and blood (E116, E123) samples. RNA-seq datasets with cohesin depletion in MCF-7 breast cancer cells and MCF-10A breast normal cells are listed in Table S4.

#### DNA methylation and somatic mutation

Differential DNA methylation was obtained from COSMIC^25^ by comparing the beta-values of cancer and normal populations for each locus using the Mann‒Whitney test. The ratio of CASs overlapping significantly altered methylation loci (p value<10^-7^) was compared to the ratio of random cohesin sites. Somatic mutations, including coding and noncoding mutations, were obtained from the COSMIC database.^25^ Conserved cohesin sites were defined as those that could be observed in over 90% of cancer and normal ChIP-seq datasets.

### Quantification and statistical analysis

Data were analyzed using Python and R. Details of specific statistical analyses are included in the main text. Statistical significance was defined as p < 0.05.

## Data Availability

• This study analyzes existing, publicly available data. These accession URLs for the datasets are listed in the key resources table. The accession number of all datasets used in this study are listed in Tables S1, S2, S3 and, S4. • This paper does not report original code. • Any additional information required to reanalyze the data reported in this paper is available from the lead contact upon request.

## Supplemental information

Document S1. Figures S1–S6

Table S1. ChIP-seq datasets used in this study, related to STAR Methods

Table S2. RNA-seq datasets used in this study, related to STAR Methods

Table S3. 3D genome datasets (ChIA-PET and Hi-C) used in this study, related to STAR Methods

Table S4. RNA-seq datasets with cohesin depletion, related to STAR Methods
