# Supplementary material for: Comprehensive multiomics analyses reveal pervasive involvement of aberrant cohesin binding in transcriptional and chromosomal disorder of cancer cells
Source: iScience. 2023 May 19;26(6):106908. doi: 10.1016/j.isci.2023.106908 (PMC10239702; doi:10.1016/j.isci.2023.106908)
Supplement: Document S1. Figures S1–S6 [file mmc1.pdf]

iScience, Volume *26*

Supplemental information

Comprehensive multiomics analyses reveal pervasive involvement of aberrant cohesin binding in transcriptional and chromosomal disorder of cancer cells

Jiankang Wang and Ryuichiro Nakato

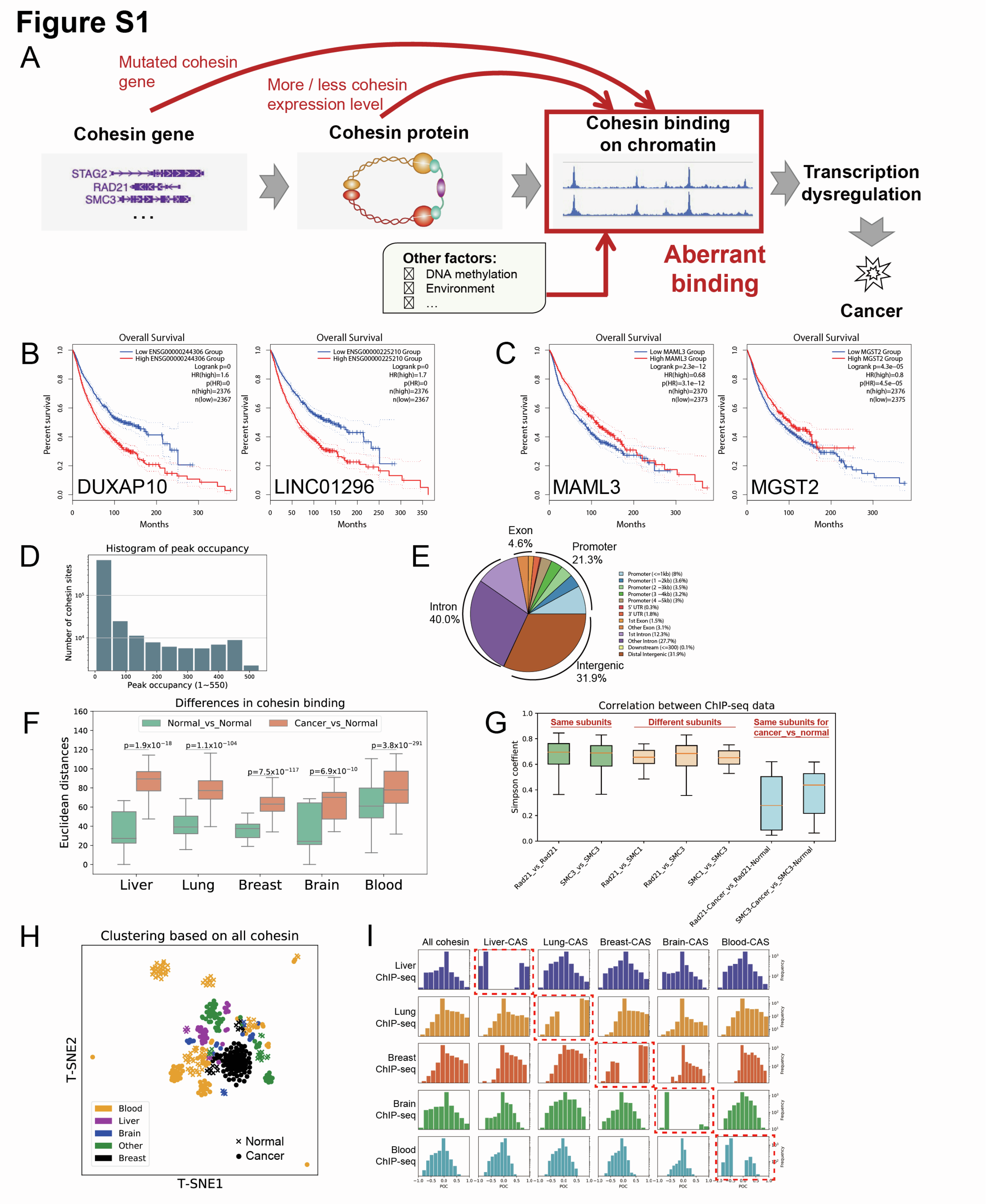

Figure S1: Concept and identification of aberrant cohesin binding in cancer cells, related to Figure 1. (A)

The concept map shows that we are primarily concerned with aberrant cohesin binding, rather than other layers. (B) Survival analysis of genes near gained cohesin sites and (C) lost cohesin sites. TCGA patient data for all cancer types was used. (D) Histogram of peak occupancy for all cohesin sites. Y-axis represents the number of cohesin sites. (E) Genomic distribution of all cohesin sites obtained from ChIPseeker. (F) Euclidean distances for cohesin binding levels between normal and normal samples and between cancer and normal samples. P values were calculated by the Mann–Whitney U test. (G) The correlation between ChIP-seq datasets with the same or different cohesin subunits. Although different subunits might introduce noise factors, they are less likely to be the primary cause for cancer-specific cohesin binding events. (H) The T-SNE plot based on cohesin binding levels at all cohesin sites. Each dot indicates a ChIP-seq sample. Tissue types and whether the cancer sample is labeled. (I) Histogram of peak occupancy change (POC) comparing cancer to normal samples. Each row represents the cohesin binding data for different tissue types. Each column represents CASs for different tissue types. X-axis: POC. Y-axis: Frequency.

Figure S2

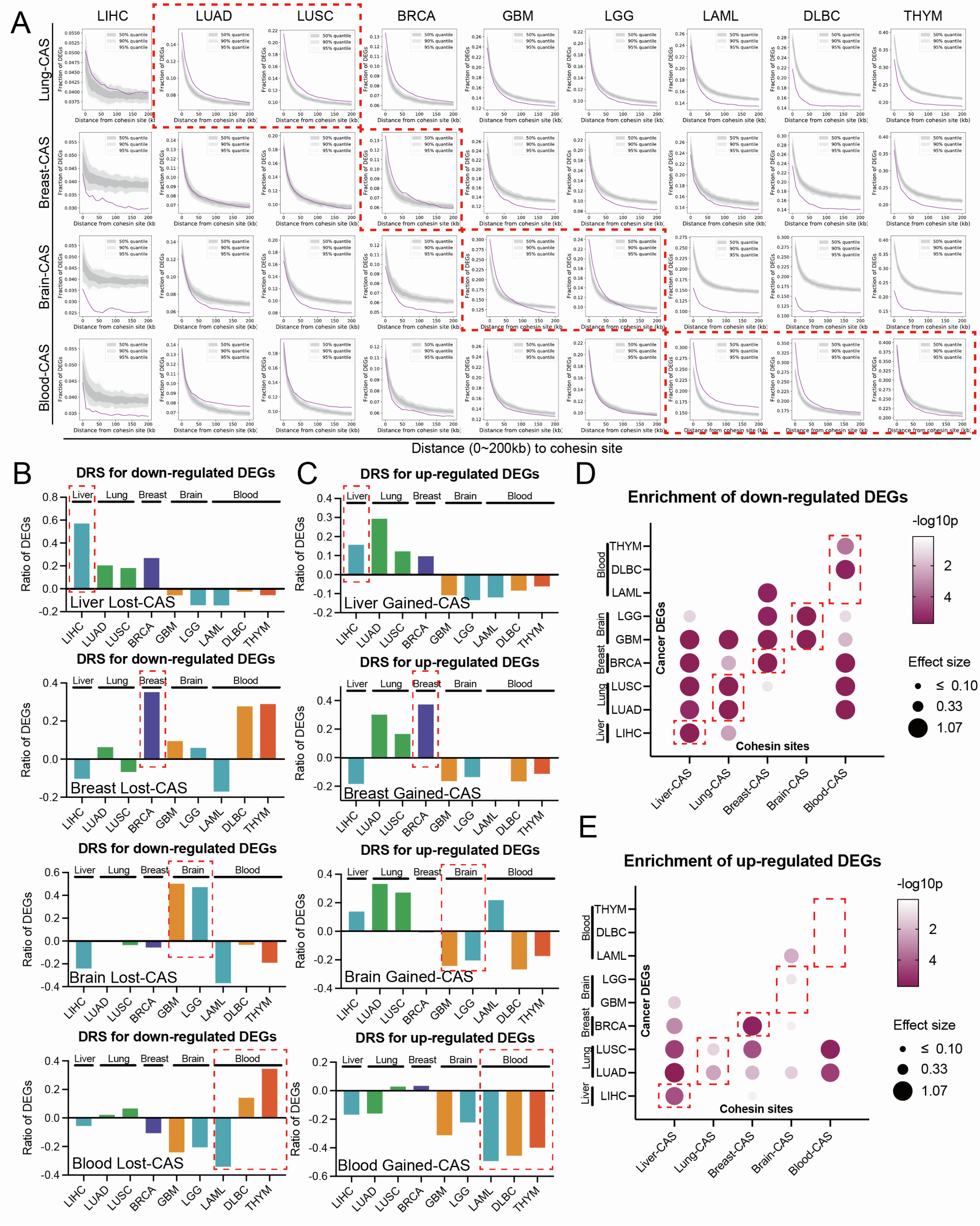
A
LIHC
LUAD
LUSC
BRCA
GBM
LGG
LAML
DLBC
THYM
Lung-CAS
Breast-CAS
Brain-CAS
Blood-CAS
Distance (0~200kb) to cohesin site
B
DRS for down-regulated DEGs
Liver Lost-CAS
Breast Lost-CAS
Brain Lost-CAS
Blood Lost-CAS
C
DRS for up-regulated DEGs
Liver Gained-CAS
Breast Gained-CAS
Brain Gained-CAS
Blood Gained-CAS
Ratio of DEGs
D
Enrichment of down-regulated DEGs
E
Enrichment of up-regulated DEGs
-log10p
Effect size
Cancer DEGs
Cohesin sites
Liver-CAS
Lung-CAS
Breast-CAS
Brain-CAS
Blood-CAS

Figure S2: Enrichment of DEGs for CASs, related to Figure 2. (A) Ratio of DEGs against distance from CASs in different cancer types. Pink line: CAS; Grey area: background model with quantiles. Red rectangles indicate the high DEG enrichment of a cancer type near the CASs of the corresponding tissue type. (B-C) DRS scores for down- and upregulated DEGs, respectively. (D-E) Enrichment of down- and upregulated DEGs at CASs by loop model. P-value and effect size were calculated by Wilcoxon test.

Figure S3

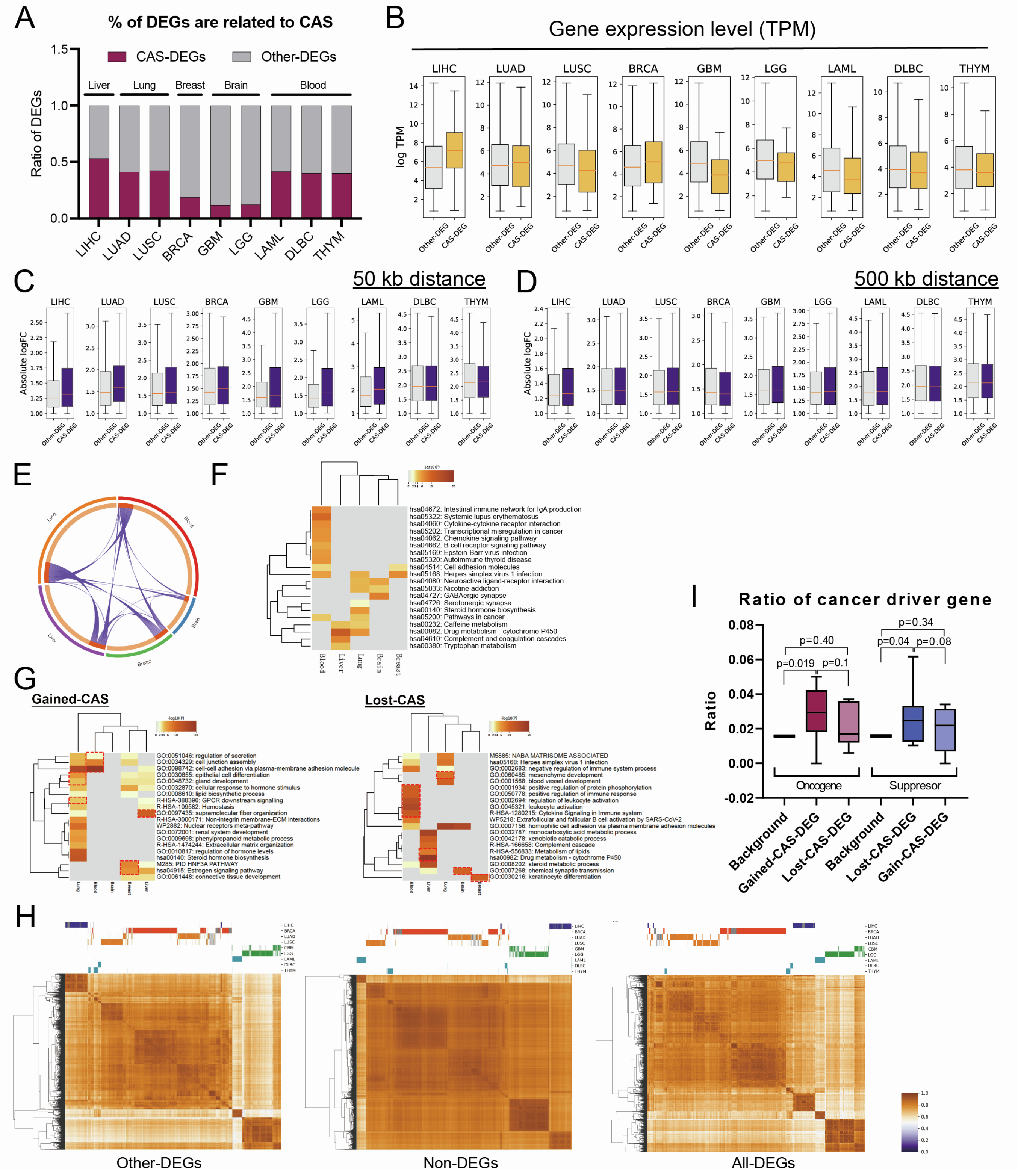

Figure S3: Characteristics of differentially expressed genes associated with CAS, related to Figures 2 and 3. (A) The proportion of DEGs within 500 kb from CASs. (B) Comparison of gene expression levels

between CAS-DEGs (5 kb from CASs) and other-DEGs. (C-D) Comparison of absolute logFC between CAS-DEGs (50 kb or 500 kb from CASs) and other-DEGs. (E) Overlap of CAS-DEGs across tissue types. (F) KEGG pathway enrichment of CAS-DEGs. (G) Ontology pathway enrichment of Gained-DEGs and Lost-DEGs separately. Red rectangles represent the pathway that are important for the indicated cancer types. (H) Clustering of patient samples based on gene expression levels of other-DEGs, non-DEGs or all-DEGs. (I) Proportion of cancer driver genes (oncogene, tumor suppressor gene) for gained-CAS-DEGs and lost-CAS-DEGs. *: $p<0.05$; one-sided Wilcoxon test.

Figure S4

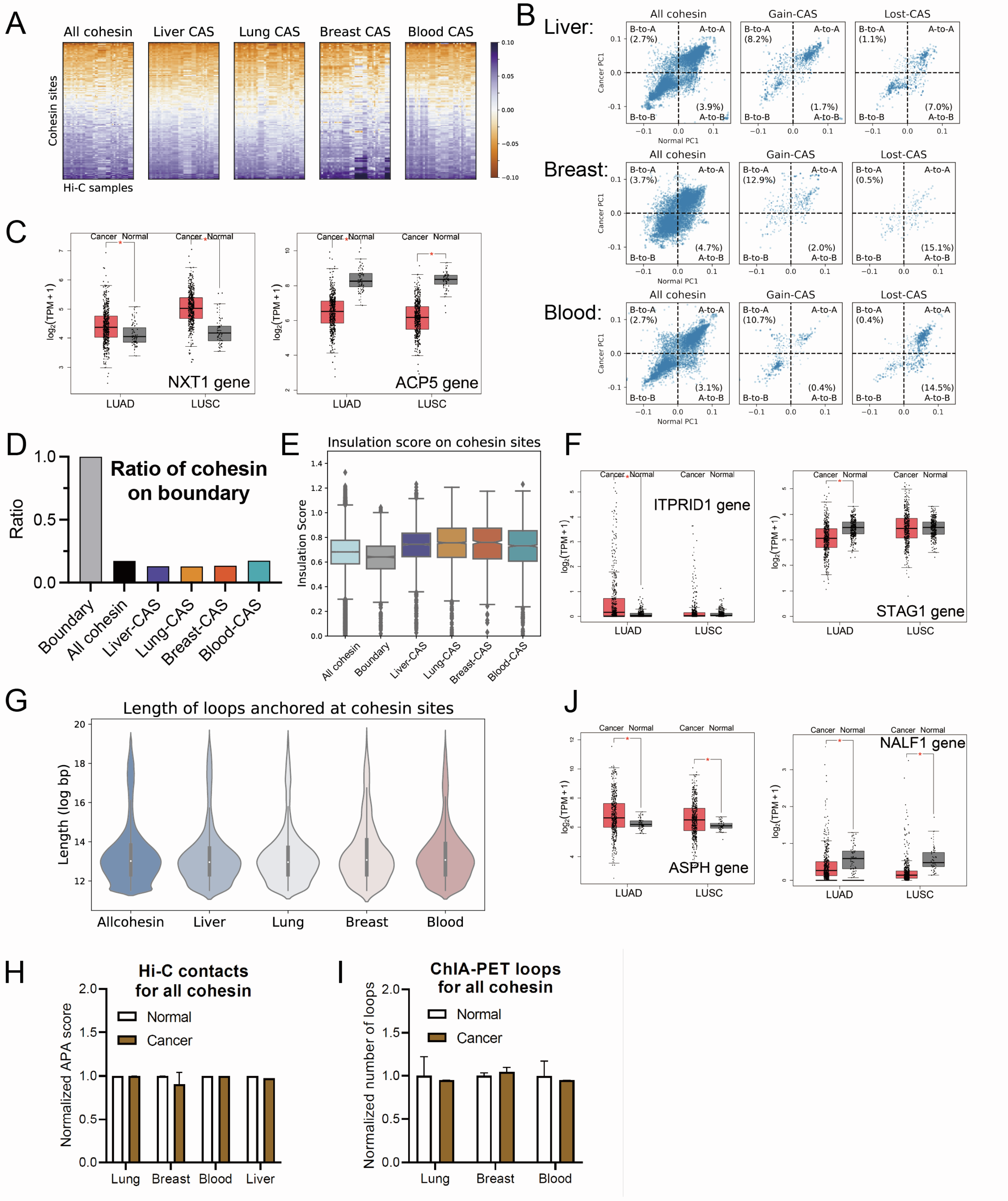

Figure S4: Alterations in chromatin structure on CASs, related to Figures 4 and 5. (A) Heatmap of

compartment PC1 values on different cohesin sites. (B) Scatter plot of compartment PC1 in normal (x-axis) and cancer (y-axis) cells. Each dot represents one cohesin site. (C) Expression levels of genes near gained or lost CASs, as shown in Figure 4C-D. *: $p<0.05$, one-way ANOVA test. (D) Proportions of cohesin sites located on TAD boundaries. ‘Boundary’ represents cohesin sites located on the TAD boundary. (E) Boxplot of IS at different cohesin sites. (F) Expression levels of genes near gained or lost CASs, as shown in Figure 4H-I. (G) Length of chromatin loops anchored from cohesin sites. (H) Normalized APA score and (I) normalized number of ChIA-PET loops for all cohesin sites. Normal samples were normalized as 1. (J) Expression levels of genes near gained or lost CASs, as shown in Figure 5E-F.

Figure S5

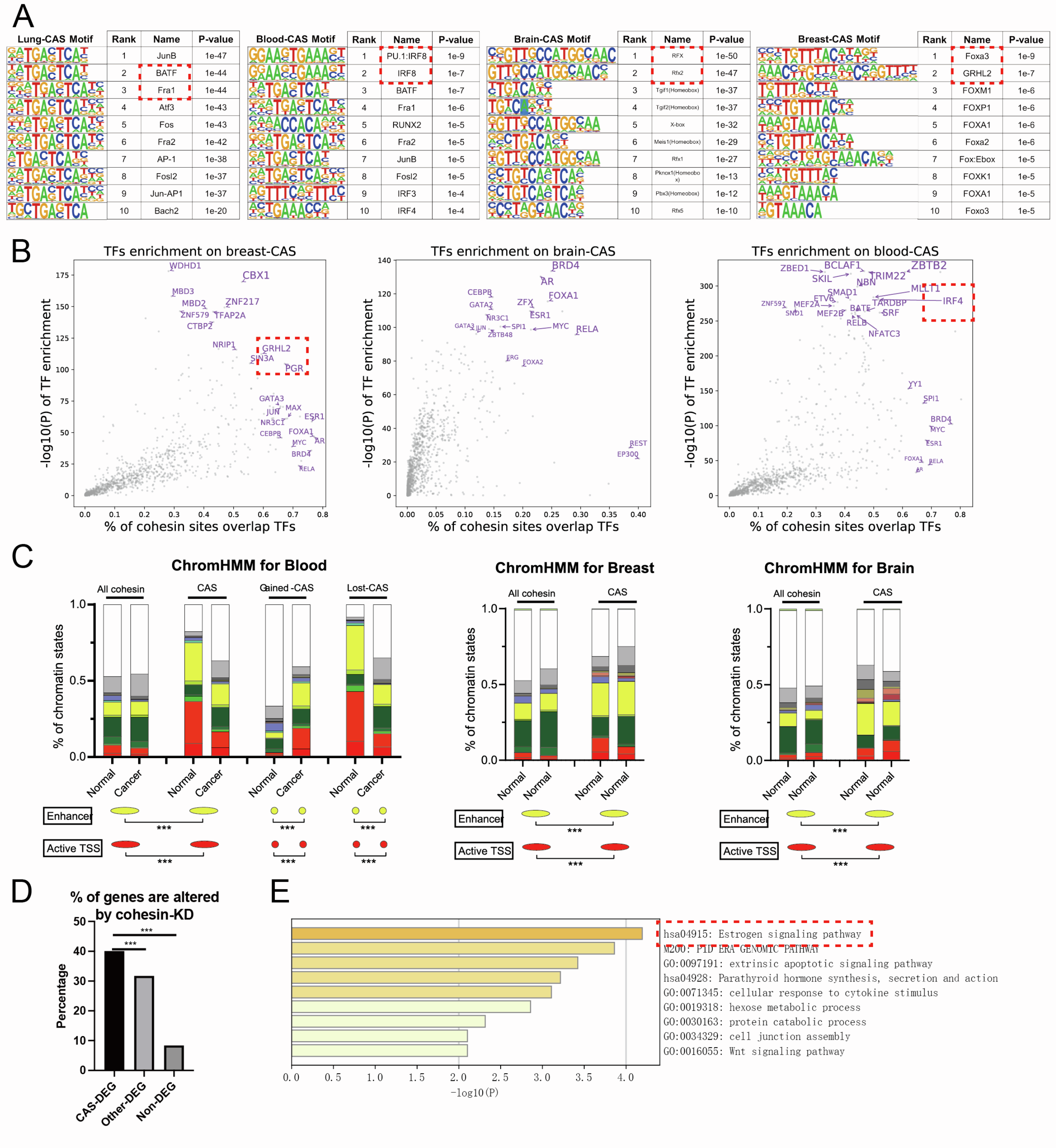

Figure S5: CASs are involved in tissue-specific CRMs of cancer cells, related to Figure 6. (A) Top 10 binding motifs for lung, blood, brain and breast CASs. Red rectangles indicate TFs known to be important for the indicated cancer type. (B) Enrichment of TF for breast, brain, and blood CASs. Each dot represents a type

of TF. The TFs in the upper right are indicated to be highly enriched at given cohesin sites. Red rectangles indicate TFs known to be important for each cancer type. (C) Proportion of chromatin states in normal or cancer samples for blood, breast and brain. ***: $P<0.001$; Fisher's exact test. (D) Percentage of genes altered by cohesin depletion in MCF-7 cells. ***: $P<0.001$; Fisher's exact test. (E) Pathway enrichment of CAS-DEGs altered by cohesin depletion in MCF-7 cells.

Figure S6

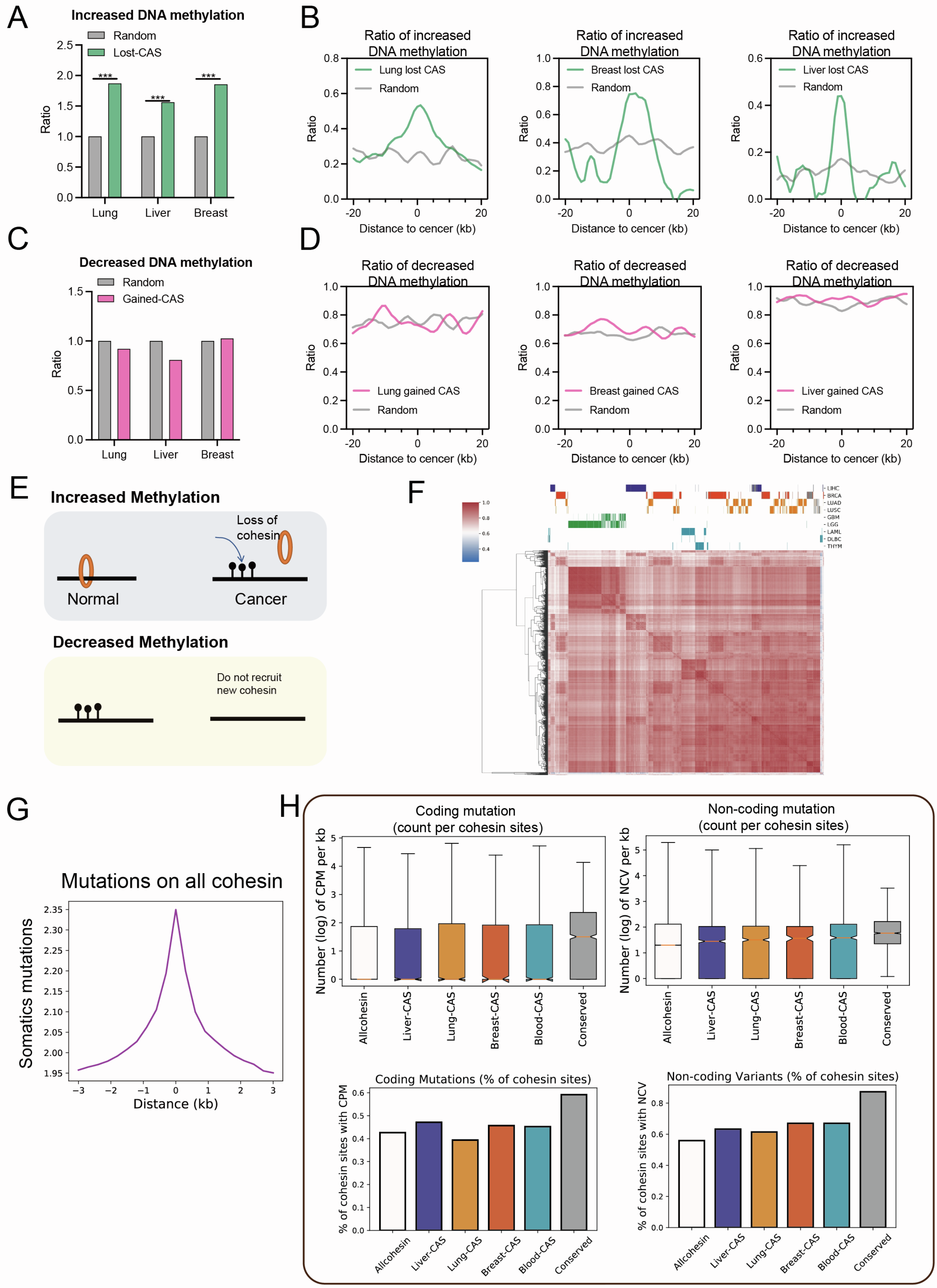

A
Increased DNA methylation
Random
Lost-CAS
Ratio
Lung
Liver
Breast
B
Ratio of increased DNA methylation
Lung lost CAS
Breast lost CAS
Liver lost CAS
Random
Distance to cencer (kb)
C
Decreased DNA methylation
Random
Gained-CAS
D
Ratio of decreased DNA methylation
Lung gained CAS
Breast gained CAS
Liver gained CAS
E
Increased Methylation
Loss of cohesin
Normal
Cancer
Decreased Methylation
Do not recruit new cohesin
F
LIHC
BRCA
LUAD
LUSC
GBM
LGG
LAML
DLBC
THYM
G
Mutations on all cohesin
Somatics mutations
Distance (kb)
H
Coding mutation (count per cohesin sites)
Number (log) of CPM per kb
Non-coding mutation (count per cohesin sites)
Number (log) of NCV per kb
Allcohesin
Liver-CAS
Lung-CAS
Breast-CAS
Blood-CAS
Conserved
Coding Mutations (% of cohesin sites)
% of cohesin sites with CPM
Non-coding Variants (% of cohesin sites)
% of cohesin sites with NCV

Figure S6: DNA methylation and somatic mutations at CASs, related to Figure 6. (A) Normalized ratios of lost-CASs exhibiting increased DNA methylation levels. *** $p<0.001$; Fisher's exact test. (B) Aggregation plot of increased DNA methylation centered on lost-CASs. (C) Normalized ratios of gained CASs exhibiting decreased DNA methylation levels. (D) Aggregation plot of decreased DNA methylation centered on gained CASs. (E) Possible explanations for the observations of DNA methylation. (F) Clustering of patient samples based on DNA methylation levels at CASs. (G) Aggregation plot of somatic mutations near cohesin sites. (H) Comparison of mutation frequencies on different sets of cohesin sites.
